# Teachers' Perceptions and Experiences of Menstrual Cycle Education and Support in UK Schools

**DOI:** 10.3389/fgwh.2022.827365

**Published:** 2022-02-14

**Authors:** Natalie Brown, Rebekah Williams, Georgie Bruinvels, Jessica Piasecki, Laura J. Forrest

**Affiliations:** ^1^School of Sport and Exercise Science, Swansea University, Swansea, United Kingdom; ^2^Welsh Institute of Performance Science, School of Sport and Exercise Science, Swansea University, Swansea, United Kingdom; ^3^Stride Active CIC (Community Interest Company), Hereford, United Kingdom; ^4^Whitecross Hereford, Hereford, United Kingdom; ^5^Institute of Sport, Exercise and Health, University College London, London, United Kingdom; ^6^Orreco Ltd., Galway, Ireland; ^7^Sport, Health and Performance Enhancement Research Centre, Nottingham Trent University, Nottingham, United Kingdom; ^8^School of Health and Life Sciences, University of the West of Scotland, Glasgow, United Kingdom

**Keywords:** menstrual health education, menstrual literacy, school, teacher, periods

## Abstract

The purpose of this study was 2-fold, to (1) explore current education provision in UK schools including barriers to menstrual cycle education and (2) assess the perceived support teachers received to deliver menstrual cycle education. Seven hundred eighty-nine teachers (91% female) from all stages of school education in England (48%), Scotland (24%), Wales (22%) and Northern Ireland (6%) completed an online survey. The survey captured information on menstrual education in schools, teacher's knowledge and confidence of the menstrual cycle, support provided to teachers, provision of menstrual products in school and perceived impact of the menstrual cycle on young people in school. Four hundred ninety-eight teachers reported lessons were provided on the menstrual cycle (63%), predominantly delivered within personal, social, health and economic or science subjects, with over half of the lessons focusing on the biology (56%) or provision of menstrual products (40%) rather than lived experiences (14%). Teachers perceived the menstrual cycle affected participation in PE (88%), pupil confidence (88%), school attendance (82%) and attitude and behavior (82%). Overall, 80% of teachers felt receiving training would be beneficial to improve menstrual education. The results highlight education is scientifically focused, with less education on management of symptoms or lived experiences. Teachers also perceive the menstrual cycle to influence multiple aspects of school attendance and personal performance. There is a need to address menstrual education provided in schools across the UK to help empower girls to manage their menstrual cycle, preventing a negative impact on health and school performance.

## Introduction

Menarche, the onset of the first menstrual bleed, occurs at an average age of 12 years old. However, the age of menarche often varies and is dependent on the interactions of genetic and environmental factors (1) and the consequential interplay between the hypothalamic, pituitary, and ovarian hormones (2). Specifically, the menstrual cycle is a repeating pattern of fluctuating hormones, primarily estrogen and progesterone, and is an example of a bio-psycho-social process; it is a normal aspect of physiology that can affect and also be affected by behavior (3). A regular and fully functioning menstrual cycle is an important marker of both reproductive and holistic health and has positive long term health implications (4). In particular, estrogen is a key regulator of bone formation (5) and also has a cardioprotective role (6). Therefore, the cyclical estrogen exposure provided through the menstrual cycle post-puberty and pre-menopause could also reduce risk of other health conditions such as osteoporosis and cardiovascular disease (7, 8), and is imperative to adolescent growth.

Additional to the impact on adolescent growth and health, menstruation and menstrual cycle-related symptoms can be disruptive and be detrimental to physical, mental and social wellbeing (4). As the cyclical process matures slowly following menarche (9, 10), disturbances such as dysmenorrhea, heavy bleeding and premenstrual syndrome can present (11). Indeed, the prevalence of dysmenorrhea in adolescent females has been shown to be as high as 93% (12). Furthermore, findings from Bruinvels et al. (13) suggests that eumenorrheic females experience on average 11 menstrual cycle-related symptoms including mood changes, stomach cramps and increased levels of anxiety and fatigue; these symptoms compromise aspects of daily life such as participation in physical activity, work capacity (14), girls' education and overall wellbeing (15). Yet research has highlighted menstrual education focuses on biology rather than addressing social and emotional dimensions, factors which may influence education and wellbeing (16).

It has been previously highlighted that girls tend to miss school, self-medicate and refrain from social interactions because of a lack of preparation, knowledge and poor practices surrounding menstruation (15). To achieve sound menstrual health, it is implied that women and girls should be able to access accurate, timely, age-appropriate information about the menstrual cycle and changes experienced throughout the life-course. Yet, a survey completed by Plan UK in 2018 (17) highlighted that 14% of girls in the United Kingdom (UK) did not feel they knew what was happening when they started bleeding, and one in four said they did not feel they knew what to do when they started their period. This demonstrates a deficit in information provided to girls in the UK about the menstrual cycle and menstrual cycle management strategies.

Alongside greater information and resources needed to support adolescent girls to confidently care for their bodies (18), an open and safe environment, free from stigma and psychological distress are also important aspects in achieving positive menstrual health. Many myths and taboos still surround the menstrual cycle. Menses tends to be associated with the need for discretion and concealment (19) and the stigmatization of secrecy and embarrassment has been further reinforced in the past by social media and adverts such as the use of blue liquid in menstrual products (17). Teachers are currently pivotal in providing both menstrual cycle education and fostering an environment for young people to learn and share experiences relating to the menstrual cycle. However, research has highlighted that in countries such as Australia, many teachers lack training and confidence in providing information and a positive environment to understand the menstrual cycle, beyond a purely biological perspective (16). In addition, many teachers report feeling embarrassed, potentially because of a lack of training received (20).

There is a growing awareness that developed countries are overdue addressing the menstruation-related needs of girls. Despite this being known, the status of menstrual education has not been investigated across the UK. There is currently a lack of understanding on what education is provided, how this is delivered, the support and guidance provided to teachers and barriers to delivery of menstrual health education. This is preventing young people developing an increased understanding of the menstrual cycle for improved menstrual and overall health. There is a great need for research in this area to better understand current menstrual cycle education, prior to implementing any new menstrual education. Therefore, this study aimed to produce a paper that provided a substantive contribution to the understanding of menstrual cycle education provided in UK schools by investigating the:

1. Current education provision in UK schools including barriers to menstrual cycle education.
2. Perceived support teachers receive to deliver menstrual cycle education.

## Materials and Methods

To address the aims of this study, an anonymous online survey was disseminated. A survey approach was deemed appropriate given the novel and exploratory nature of the research (21). Convenience sampling with emails sent directly to schools and snowball sampling using social media platforms were used to target teachers in the UK. The aim of this study was to achieve a breadth of understanding of teachers' experiences in this area, therefore, convenience sampling was considered valid and reliable in the context of this research (22). The inclusion criteria were UK based qualified teachers, from all subjects, year groups or levels (school systems defined as year groups within England, Wales and Northern Ireland and equivalent matched as levels within Scotland, e.g., P2 first level in Scotland is equivalent to year 1), and type of schools (including state, independent, mixed and single gender schools). Following receipt of institutional ethical approval (approval number NB_2020_041b), participants were required to provide consent by accepting all information provided before completing the survey. The survey was launched 5th November 2020 and was open for 16 weeks (closed 28th February 2021). A total of 789 responses were included in the final data set. IP address analysis was completed to check if any individuals completed the survey more than once, according to the CHERRIES checklist (23).

An online survey was created (JISC online surveys) to identify menstrual cycle education in schools across the UK and provided the platform to securely store all data. A pilot study established content and face validity (*n* = 39); minor amendments were made to question order following this. Data from the pilot study were not included in the final data set due to amendments in the survey affecting results. The final survey was estimated to take 8 min and consisted of a maximum of 48 questions; logic was applied to the survey to ensure only relevant questions were completed. Survey questions were written in common language to cater for teachers of all subjects and included country, local authority/council, school type, year group(s)/levels, subject(s) taught, gender, age, and teaching experience (years) to enable data slicing. Further questions focused on lesson provision (e.g., content, frequency and audience), students' access to menstrual products, barriers to education and perceived support provided to teachers. Questions were multiple choice, perception-based responses quantified using four and five-point Likert scales ranging from “no impact at all/not at all confident/very uncomfortable” to “extreme impact/extremely confident/very comfortable,” or free text answers.

The raw data of a subset of questions was exported from JISC directly to Microsoft Excel software or Jamovi software (version 1.6.23.0) to be analyzed. Descriptive statistics, reported as frequencies and, associations between countries, school type and primary and secondary school teachers were determined using chi-square analysis with statistical significance set at a value of *X*^2^ = <0.05. Free text responses were analyzed using qualitative description (content analyses) by the first author and were questioned by another member of the research team. Counting frequency of words directly in text was completed for some free text responses or descriptive codes were assigned to data to identify raw data themes, this allowed for interpretative codes to be generated. These codes grouped descriptive codes into more abstract concepts. Pattern codes were identified which recognized relationships between interpretative codes.

Although the figures and percentages noted in this study are based upon the total number of participants who responded to the specific question, it should be noted that more than one answer may have been possible; this is annotated in the relevant tables.

## Results

### Participant Characteristics

Responses were predominately from female teachers (91%), aged between 21 and 59 and taught at state schools (91%) across the UK with 56% having over 10 years of teaching experience (*n* = 446). Respondents were located across all nations; England (48%), Scotland (24%), Wales (22%) and Northern Ireland (6%) and were represented from primary/early/first level (47%, *n* = 373) and secondary school (56%, *n* = 445) from all curriculum subject areas (Table 1), including 40% mixed/primary, 22% physical education (PE) and 11% science.

**Table 1:** Participant demographics (*n* = 789).

**Characteristics**	**Percentage (%)**
**Country**	
England (37/40 counties)	48
Wales 17/22 counties)	22
Scotland (29/32 counties)	24
Northern Ireland (5/6 counties)	6
**Age**	
<21	0.5
21–29	22.9
30–39	35.1
40–49	26.6
50–59	13.6
60+	1.3
**Gender**	
Male	9
Female	91
**Teaching experience (Year's)**	
<1 yr	6
1–3 yrs	12
4–9 yrs	25.5
10+ yrs	56.5
**Type of school**	
State	65.1
State (academy)	26.3
Independent	8.6
**Subject's taught**	
Mixed (primary/early/first level)	40.7
Mathematics	4.4
Science	11.4
English	5.6
Geography	2.4
History	2.6
Physical education (PE)	22.5
Design and technology	1.9
Information technology (IT)	1.5
Art	1.2
Music	0.5
Modern foreign languages	1.7
Multiple subjects	3.8
Class contact cover	1.5
Senior leader	4.4
Personal, social, health and economic (PSHE)	10.8
Other (Welsh, Welfare, Religious Education, Ethics, Drama, Support for learning, Modern studies and social studies, Citizenship, Business, health and social care)	12.8

### Menstrual Cycle Education Delivery

**Figure 1:**
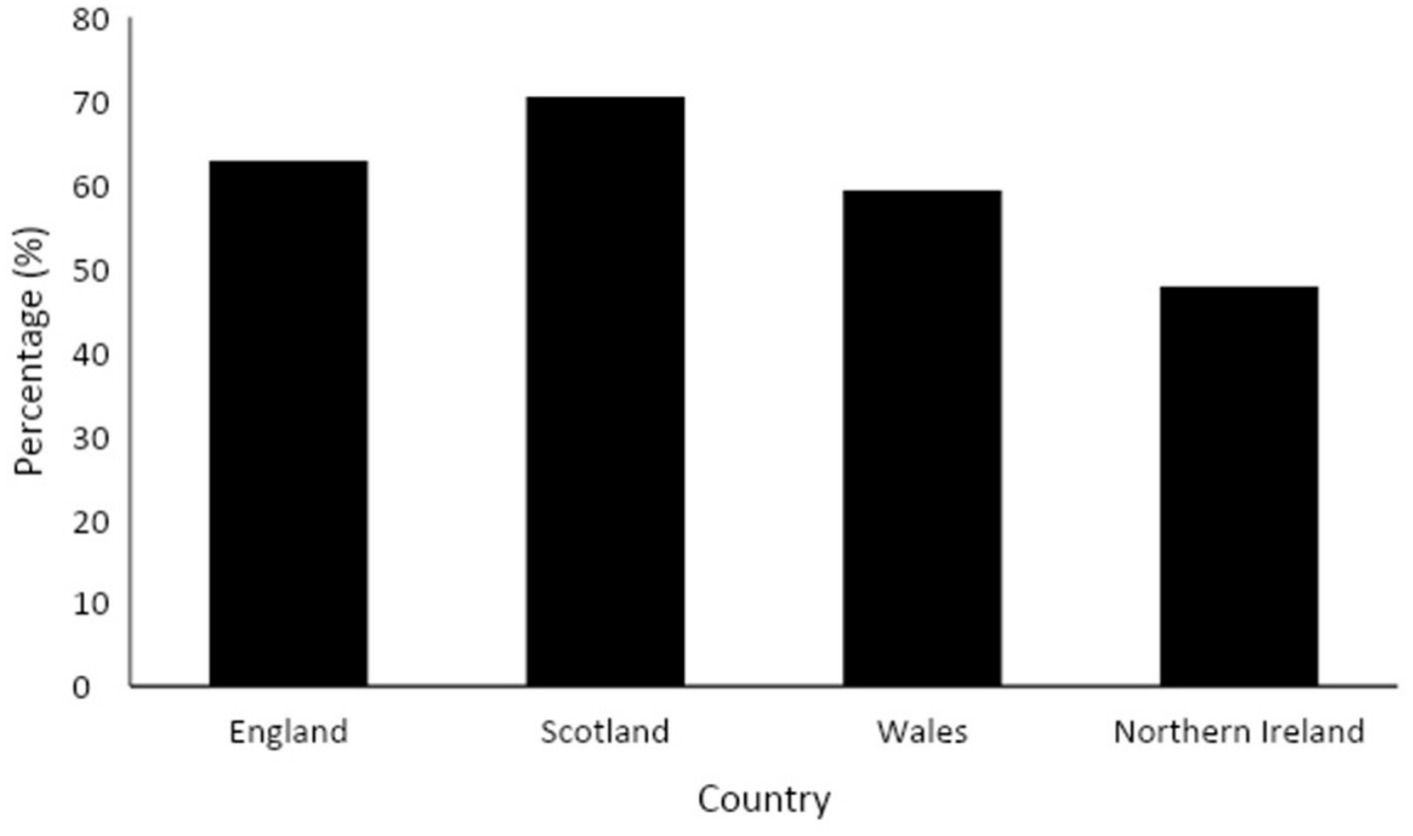
Percentage of lesson provision reported by teachers within each country (*n* = 789).

Provision of education relating to the menstrual cycle was reported by 63% of respondents (*n* = 498); Twenty ninety percentage reported they did not know if lessons were provided (*n* = 230). This was consistent between state and independent schools [$X_{\left(2\right)}^{2}$ = 2.26, *p* = 0.323], but varied between primary and secondary schools (Table 2) and across countries [*X*^2^ = 28.8, *p* < 0.001) (Figure 1).

**Table 2:** Comparison of lessons, product provision and teacher delivery of menstrual education in primary and secondary schools across the UK.

	**Primary school (%)**	**Secondary school (%)**	**Statistical comparisons^a^**
**Lesson provision reported**	74	53	$X_{\left(2\right)}^{2}$ = 43.5
England	48	51	*p* < 0.001
Scotland	77	22	*n* = 758
Wales	37	61	
Northern Ireland	36	68	
1–2 lessons in one academic year	52	41	X > 0.05
**Provision of educational content**			
Yes	81	68	$X_{\left(1\right)}^{2}$ = 7.57
No			*p* = 0.006
Don't know			*n* = 279
**Comfort of teaching**			
Very comfortable	39	56	$X_{\left(1\right)}^{2}$ = 7.57
Comfortable			*P* < 0.001

a *Chi-squared statistical analysis results to determine associations between primary and secondary school data*.

Time and support were most frequently reported reasons for absence of menstrual cycle education, although lack of support, resources, COVID-19 and parents were also commented on. Teachers from primary schools stated issues such as “(the) leadership team don't see it as important,” “(there is) limited time to fit into the curriculum,” “parental reaction, some (parents) would be mortified” and “COVID restricting ability to split boys and girls to teach lessons” as reasons for lessons not being provided. Similarly, secondary school teachers reported issues such as “time and it isn't seen as a focus,” but also cultural factors and knowledge levels were described “my school is located in a predominately Asian area where these matters are not discussed,” “It's a Catholic school so that impacts upon decisions” and “lack of knowledge for teachers.”

The most reported barriers preventing change or difference in approach to delivery of menstrual education were also lack of time (40%), teacher confidence/resources (35%) and parents (9%); additional comments included “staff seem too embarrassed outside our department to discuss it, they seem to find it daunting and avoid teaching it,” whereas another teacher summarized “time, training and parental concerns” as the restricting factors. Despite multiple reasons and barriers affecting menstrual education, there were some positive comments from teachers reporting there were no barriers and “Menstrual education is informative and well-supported for children in our school.”

**Figure 2:**
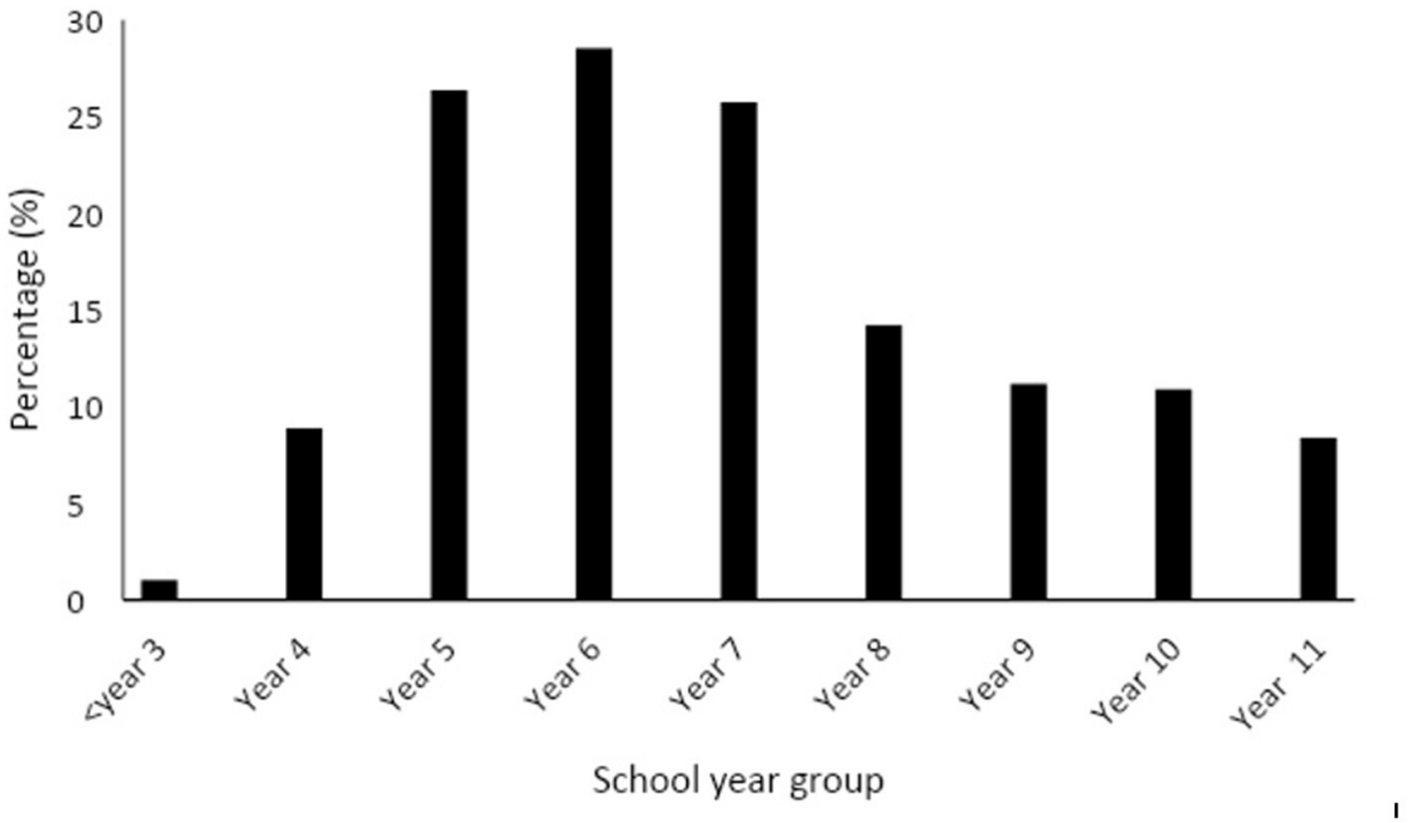
Percentage of lessons provided across year groups at school. Scotland has been matched to equivalent year groups in England, Wales and Northern Ireland.

When asked about which year groups received menstrual cycle lessons; Seventy nine percentage of responses indicated that lessons were taught from 5/P6 to year 7/S1, Figure 2 provides data displayed for each school year group. Of teachers who were aware of their school's menstrual cycle syllabus, 144 teachers reported that a maximum of two lessons were provided within one academic year; this is consistent across primary and secondary respondents (Table 2). Comments from teachers displayed perceptions that this was the total amount of education, rather than per academic year as summarized by one response, “Too often it is a one-off lesson.” In addition to frequency, teachers expressed concerns that education was not delivered early enough; “I think it needs to start at a younger age because girls can start their period before P6,” “It has to start earlier- definitely in primary school.” One teacher confirmed, there is “Definitely not enough education around this topic in school, it is very limited...it's a big topic that needs more attention.”

Over one-third of respondents noted PSHE and biology as the principal subjects for menstrual cycle education. Over half (56%) of education focused on biology of the menstrual cycle and 40% on menstrual products. Topics not frequently covered within menstrual cycle education were the benefits of exercise on menstrual symptom management, with only 18% of respondents providing this information in school. In addition, irregular menstrual cycle and symptoms (9%) and what to do if experiencing irregularities (7%) were not often taught or discussed. One teacher shared, “I think it is essential for schools to give children more information on what a “normal” and abnormal menstrual cycle might look like and when to seek further help or advice from a (General Medical Practitioner) GP.”

Most countries and school types reported education was predominantly provided to mixed girls and boys, but there was a higher tendency in Northern Ireland (36%) and independent (33%) schools to teach girls only compared to <25% in other countries and state schools. One teacher shared, “I like the balance of delivering all information to a mixed gender group whilst providing opportunity for further discussion and openness in single gender group. Pupil feedback showed strong support for this strategy.”

### Confidence and Support to Deliver Menstrual Cycle Education

Over three-quarters (76%) of participants reported educational content was provided to them to deliver menstrual cycle education. Seventy-eight percentage of state schools were given educational content, whilst this was lower in independent schools (54%) [$X_{\left(1\right)}^{2}$ = 7.54, *p* = 0.006]. A difference was also evident between primary and secondary schools (Table 2), but there was no variation between countries [$X_{\left(3\right)}^{2}$ = 0.227, *p* = 0.973]. Three-quarters (75%, *n* = 213) of respondents reported they felt supported to teach menstrual cycle education, which was consistent between state and independent schools [$X_{\left(2\right)}^{2}$ = 1.07, *p* = 0.587]. Comments such as “I feel that I have the right resources to help teach this topic” contributed to feeling supported to delivering menstrual education. With respect to comfort of teaching, 47% of participants felt comfortable (*n* = 136) delivering lessons, whereas 23% were not comfortable teaching information about the menstrual cycle. Teaching experience (<10 or >10 years teaching) did not influence comfort of teaching [$X_{\left(4\right)}^{2}$ = 3.13, *p* = 0.537], although individual perceptions and confidence varied, it was shared “I feel I know enough to teach it but having never done it before, I would be a little anxious,” whilst “Experience in delivering content over the past few years” was noted as being beneficial.

Overall, participants felt more confident *talking* about the menstrual cycle (40% confident) than *knowledge* of the menstrual cycle (34% confident). Teacher comments highlighted gender was an influencing factor on confidence, such as: “As a male I do not feel confident on this topic” compared to comments such as: “As a woman, I feel quite confident being able to teach this.” The impact of gender was also echoed when referring to the audience: “We deliver the same content to boys and girls and the boys sometimes feel uncomfortable, making it a little more difficult.” Generally, it was shared that delivery could be difficult irrespective of students' gender: “It is always a difficult subject to teach due to the shyness of students, however by raising awareness that it's perfectly normal (it) should encourage girls to speak up/out about periods.” Although one teacher shared, “As a female teacher in a girl's independent school, I feel comfortable delivering the content for the girls we teach because the more comfortable we seem then the more comfortable the girls will be to learn and engage with the content.”

It was common for individuals to reflect on their own personal experience of the menstrual cycle and how this influenced confidence and delivery of education, however this was not always from a positive perspective in which feelings of embarrassment prevented student support and highlight a lack of staff support:

As a woman who has heavy periods that has struggled to get through teaching a lesson without leaking and has leaked all over my clothes before a lesson, I am sympathetic. If the teacher hasn't experienced heavy, painful periods then there is a lack of understanding of the effect on the child who is still learning. Sometimes I wish it was acceptable for me to teach wearing a heat pack, but I'm too embarrassed.

Subject specialism was an influencing factor on confidence; 81% of respondents specifically reported they taught the science of the menstrual cycle; one teacher stated, “I am a science teacher, so I teach about this already.” The age of pupils being taught also altered confidence levels: “(I am) Confident to teach Y6 but this is my first year in Y4 so not so sure about the younger age group.”

**Figure 3:**
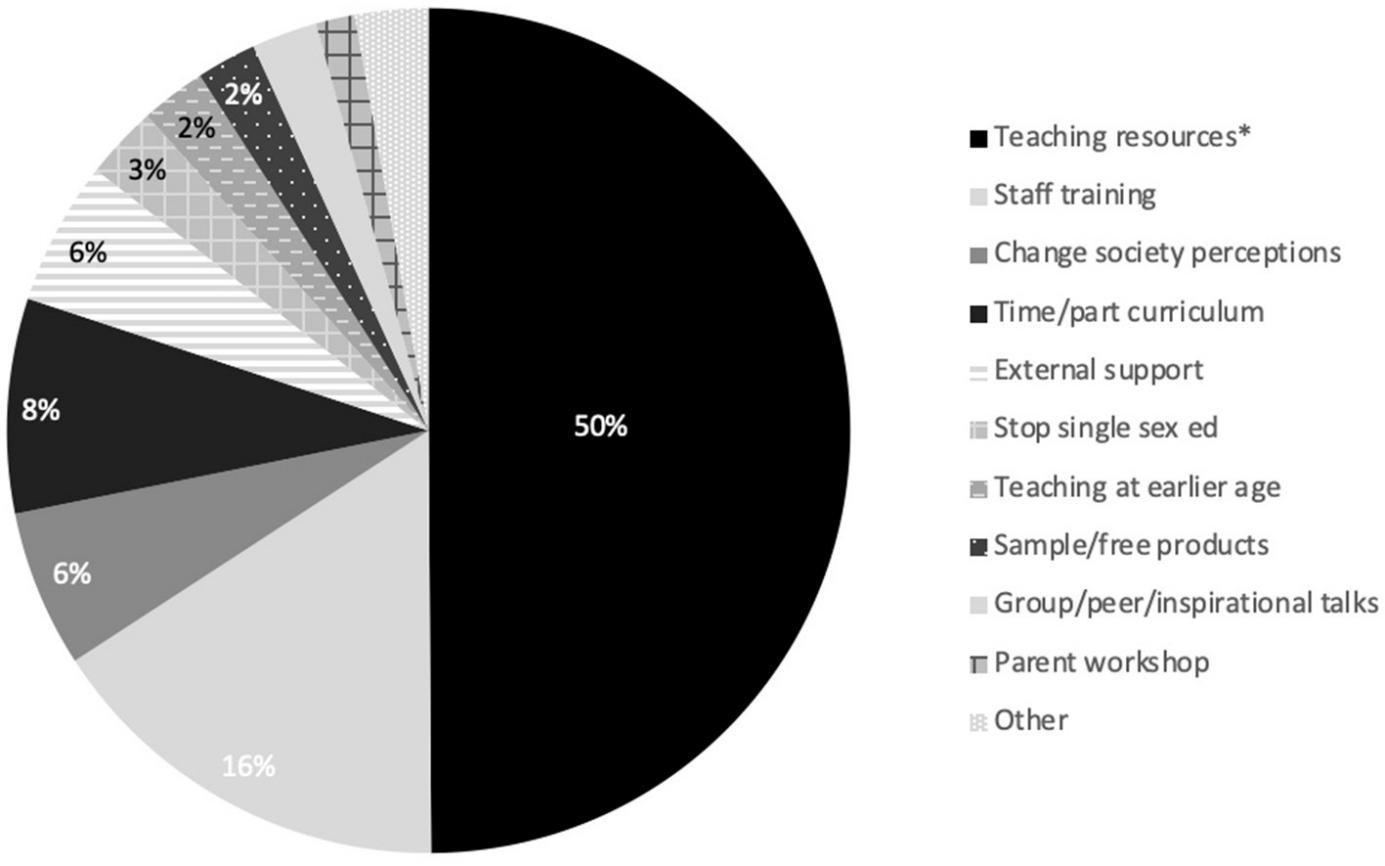
Perceived approaches to improve menstrual cycle education, listed in order of most frequently (*indicates most frequently reported) to least frequently reported (*n* = 598).

In addition to the aforementioned key themes affecting confidence and comfort (personal experience of the menstrual cycle, experience teaching, gender and student age), of important note was also the frequent comments relating to training and resources. One teacher declared that they have “never undertaken any CPD (continued professional development) or been advised in any way about delivering this content which makes me feel less confident in teaching it.” Teachers also reported they “did not feel comfortable,” “find it daunting and avoid teaching it” due to a lack of teacher training. There were some instances when teachers reported receipt of training, “Our school has provided information and training.” However, circumstances of lockdown and remote learning resulting from COVID-19 further impacted teacher training, with staff missing training because of the pandemic; one participant summarizes: “Due to COVID-19 we have been unable to deliver staff training as per normal.” Most participants (80%) selected training specific to teaching content/providing education on the menstrual cycle would be beneficial, this was consistent across state and independent schools [$X_{\left(1\right)}^{2}$ = 0.153, *p* = 0.696] countries [$X_{\left(3\right)}^{2}$ = 1.20, *p* = 0.753] and primary and secondary schools [$X_{\left(1\right)}^{2}$ = 1.96, *p* = 0.162]. Teaching resources were most frequently reported to improve menstrual education provision (Figure 3). The most desired format of resources was e-learning webinars (Table 3). “More modern video, age-appropriate content and leaflet or booklets provided for girls to take home and discuss with their parents would be beneficial”—age-appropriate resources were also highlighted as being needed and resources available in different languages, for example, the Welsh language. In addition, resources which are appropriate for students with special educational needs would benefit teachers. One respondent shared: “I have taught menstrual cycle as part of the Sex Education curriculum over the last few years. This year, I have been provided with a range of resources on the menstrual cycle from the new curriculum. This has really helped with teaching and to provide high-quality teaching.” Specifically in England, 57% of participants reported information was provided in the new curriculum to support the delivery of menstrual cycle education, with 39% reporting information and delivery was different because of the new curriculum guidance which created some uncertainty. However, others noted that they were “Unsure of the new guidance” and “it's (the new curriculum is) new so (it is) difficult to say.” One teacher shared, “it (menstrual cycle education) has been delivered before but no guidance has been provided as part of the new curriculum.” With respect to confidence, 43% of participants felt confident and comfortable delivering the new curriculum content and 35% reported to be extremely confident and comfortable.

**Table 3:** Suggested formats of requested teaching resources to enhance menstrual cycle education (*n* = 457)^a^.

**Type of resource requested**	**Percentage of responses (%)**
Lesson plans/schemes of work	16.1
Videos	12.8
Handouts	11.9
Tasks/demonstrations for products	11
Age appropriate	8.7
Handouts for parents	7.3
Experiences	6
Power points	5.5
Posters	5.5
Universal to all	4.1
Online	4.1
Visual	2.8
Appropriate for special educational needs	1.8
Available in Welsh	1.8
Webinars	0.9
Government devised	0.5
Social media for pupils	0.5
Interactive	0.5

a *Number of respondents that answered the question*.

Other areas of support which teachers perceived would help delivery of the subject, included parental support, whole school policy, time, and gaining external support which in some instances have been removed “we no longer get the nurse visits we used to,” which are felt to be fundamental “schools should be working with health—the onus on health. Schools should facilitate it, not coordinate it.”

### Menstrual Product Provision

The availability of free menstrual products within school was acknowledged by 72% of participants, this varied based on type of school [state 74%, independent 50%—$X_{\left(3\right)}^{2}$ = 18.8, *p* < 0.001], primary and secondary (Table 2) and country with Wales reporting the highest provision of menstrual products within school (84%) and Northern Ireland the lowest (50%). Menstrual products are most frequently provided by staff, although in some instances resources were sourced from external organizations as shared by one teacher: “We have registered with the charity Red Box and have had their resources in place at school for a while now.” External funding has also been sought to support schools to provide products, “Our school is in a very deprived area and therefore the Period Poverty grant has enabled us to stock up to have enough provisions available at all times.” Products were commonly located or accessed through staff including student services, classrooms, or reception. It was noted this was due to behavior; menstrual products are “Offered to pupils for free but pupils abuse this and often vandalize bathrooms with said products quite badly.” Yet many teachers felt the need to request access to menstrual products affected provision, as summarized by one respondent “To access these (menstrual products) children have to go to the office to ask, which they are often embarrassed to do.” However, this response was not consistent and examples of proactive messaging and access to menstrual products were noted: “Promotion in (my) school is very good, posters (are) up in key places which tell girls where products are available (in several places) and regular reminders (are sent) to staff via emails.” Furthermore, examples of student-to-student peer support were provided: “Senior girls do presentations to everyone re (regarding) availability and where to access them (in all toilets and support areas, plus in PE)” and “We have a very active girls group prompting period hygiene and trying to break the barriers.”

### Perceived Impact of the Menstrual Cycle

The menstrual cycle was perceived to affect multiple areas. Specifically, 88% felt PE was affected with it being noted that “Some will not participate (in physical education) when menstruating. If they do participate, they can be less enthusiastic.” Encouragement to continue participating in physical activity was frequently sport dependent “The only time we allow girls to be exempt from PE is if they are swimming.” In addition, teachers perceived the menstrual cycle to affect pupils' confidence, “their confidence and mood can be effected” (88%); school attendance “(the) main impact is on attendance which leads to missing work” (82%); attitude and behavior (82%); learning (64%) and exam results (45%) with one teacher summarizing “reduced engagement, increased non-participation, reduced energy levels and effort in class (and), reduced confidence.” When compared between state and independent schools, attitude and behaviors (state 82%; independent 76%) and confidence (state 88%, independent 83%) were similar. Respondents also commented on relationships, self-esteem and “focus in class” being affected by the menstrual cycle. A contrasting view of “it (the menstrual cycle) doesn't affect anything” was shared, yet this does not represent the majority of participant responses which are summarized by one participant stating the menstrual cycle affects “All aspects of learning and life.”

## Discussion

The purpose of this study was 2-fold, to explore (1) current education provision in UK schools including barriers to menstrual cycle education and (2) perceived support teachers receive to deliver menstrual cycle education. Menstrual education had not previously been quantified across the UK, therefore, the key intention of this research was to engage with teachers within UK schools to understand their experiences of menstrual education alongside identifying provision of resources, confidence and comfort levels to teach. Overall, the findings of this study highlight the urgent need to address menstrual cycle education in schools across the UK by ensuring all young people have access to menstrual cycle education.

The need for greater menstrual health education was evident in our findings as 37% of respondents reported that menstrual education was not provided, or they were unaware if it was taught at their school. This increased to almost half of teachers in secondary schools. Teachers also reported education needs to be delivered at a younger age and occur on more than one occasion, as teachers in our research frequently reported just one or two lessons were provided in year 6 (or equivalent across countries). Support is required for teachers to improve delivery of menstrual education through provision of resources, training and timetabling. Indeed, the greatest barriers to preventing a change or increase in provision of menstrual education was lack of time (40%) and teacher confidence/resources (35%). This is consistent for all countries, school type and level, despite the new curriculum obligation in England. However, the new curriculum was only implemented in September 2020, with the current survey initiated in November 2020 and also coinciding with COVID-19. A combination of timeline and COVID-19, as reported by multiple respondents, may not have provided sufficient time for the new curriculum obligation to come to fruition. It is important to note that during the time of COVID-19, with home schooling enforced, there is a group of young people with significantly less menstrual education than previous years and therefore a heightened gap in education.

Despite growing recognition of menstruation being an important global public health issue (24–26), many gaps still exist in the evidence for informing programme and policy (4). Recently, investment has seen considerable improvements in education metrics across low-and middle-income countries. However, from our findings, high-income countries such as the UK are yet to improve menstrual education within schools and support teachers to achieve this. Menstruation has emerged as a neglected, but significant obstacle to education (18), extending to health, dignity, psychosocial wellbeing, employment and participation in society (27, 28) through a lack of menstrual cycle education. Findings from the present study identified teachers felt aspects such as PE (88%), confidence (88%), attendance (82%) and attitude and behavior (82%) were affected by the menstrual cycle, especially around the time of menstruation. Bush et al. (29) identified 27% of students from New Zealand sometimes or always missed school due to menstrual symptoms, whilst studies conducted in Australia and Taiwan reported that adolescent girls with dysmenorrhea had poorer achievement in school than their peers (30, 31).

Many girls across the world enter puberty with knowledge gaps and misconceptions about menstruation, therefore being unprepared to cope with it and unsure of when and where to seek help (14). Reports have suggested adults, including parents and teachers, are ill-informed and uncomfortable discussing reproduction and menstruation (14), highlighting an ongoing deficit in knowledge. This was evident within our findings; <1-half of participants feeling very comfortable teaching about the menstrual cycle. There was also a focus on the biology of the menstrual cycle (56%) taught within science lessons or menstrual product information (40%), whilst teaching aspects which would help girls manage their menstrual symptoms or highlight areas which are important for general health was limited. For example, abnormal uterine bleeding is a common problem which has significant adverse effects on affected adolescents' quality of life (32), usually leading to a state of anxiety (33). However, while often idiopathic, it may also be the first sign of underlying bleeding disorders, consequently, it is an important symptom for adolescents and their parents to be aware of (33). This is often more prominent in adolescence given the high percentage (37%) of young women who experience heavy menstruation (34). Additional clinical conditions such as endometriosis can also cause anxiety and depression and can be a disabling condition, compromising social relationships and mental health (35, 36). However, given these clinical conditions can be difficult to diagnose it is essential that young people understand the conditions and symptoms of both regular and irregular menstrual cycles. This type of information would improve the likelihood of girls being able to manage their menstrual cycle and seek appropriate help where needed. In turn, it could up in reducing the negative impact on health and wellbeing and reduce the negative consequences related to school attendance and participation in physical activity. Crucially, this requires support for teachers to increase their confidence and knowledge to deliver this information and for education to be extended beyond the science of the menstrual cycle, considering health, social and emotional dimensions.

Despite many challenges existing in achieving quality menstrual education for all, 80% of respondents in this study perceived that professional development and further training would be beneficial to them to enhance menstrual education. Training should be provided to all teachers to ensure they have an awareness of the menstrual cycle and the potential impact on attendance, attitude, confidence and academic achievement. Teachers within this study considered e-learning webinars and reading resources to be the best formats for further training and development needs. Increased teacher support and improved menstrual health knowledge would enable future research to focus on the efficacy of menstrual health education interventions within schools. This has been evidenced in low-and middle-income countries where education interventions have improved knowledge, awareness and menstrual cycle management in young people (15, 30). For instance, in Uganda, those in schools receiving menstrual cycle education interventions were more at ease discussing menstruation, with support from peers, teachers and family being an integral part to the girls' understanding and openness to talk about the menstrual cycle (37). Armor et al. (38) reported 48.8% of the young women who completed a 3-month web-based menstrual education intervention had increased awareness of what constituted a “normal period” and two-thirds had improved their understanding of the menstrual cycle (38).

Both personal experience and gender should also be considered when implementing teacher support to improve menstrual education, as conveyed within our results. Both male and female teachers should complete training to increase awareness of the impact of the menstrual cycle on school attendance and performance, along with advice on how to initiate conversation for boys and girls. It should not be assumed, due to personal experience, that all female teachers are comfortable teaching menstrual education. There was disparity in testimonials, in which some felt personal experience assisted the ability to teach, whereas others felt it was not acceptable to share experiences of heavy bleeding and leaking and were too embarrassed. This is consistent with previous research which reported that many teachers felt embarrassed, potentially resulting from a lack of training received (20). Similar findings have been reported by Brown and Knight (39) within sport; the variation in female coaches' personal experiences of the menstrual cycle limited their overall understanding and awareness as their own experiences are not universal. This impacted on the support provided to female athletes, in combination with additional perceptions of secrecy relating to the menstrual cycle, reducing the informational and emotional support participants provided.

In addition to informational education, menstrual product provision was explored within the current study. The results detailed teachers in Wales reported the highest provision of menstrual products, this is surprising given the Scottish government bill starting in April 2019 for all schools to provide free menstrual products and a similar approach in England. However, since data collection this may have changed as Scottish Government did not pass the bill until January 2021, to legally provide free menstrual products. Many teachers reported there were products available, however they were frequently kept by individual members of staff which other teachers lacked awareness of and subsequently would not have made them readily available for young people. Schools, through the support of teachers, should make provision of free menstrual products more widely known and accessible to young people which may also assist with attendance and participation in PE.

Consistent with recommendations that we need to create a society in which menstruation is less stigmatized (40), teachers need to address their own discomfort when discussing and teaching menstrual education. Part of this process involves all teachers and pupils receiving education. However, it is important to note that education in schools is only one aspect in achieving a cultural shift. Other factors such as parental support and appropriate media depiction of the menstrual cycle is paramount in order to normalize conversations within society and freely communicate about menstruation.

Limitations of the study should be considered; our results are limited in the percentage of male compared to female respondents. Within the UK, statistics state that the total number of teachers are 30% male and 70% female (41). Within our sample, 91% of respondents were female. This unequal response between male and female teachers could be due to the menstrual cycle still being seen as a taboo topic within society and male teachers perceiving this not to be an area related to them, or schools allocating female staff to teach this area of the curriculum as they may perceive females as being more familiar with the menstrual cycle due to their lived experiences. However, this is speculation and we cannot be certain this is the case within our sample. Furthermore, responses to the survey may not be fully representative of all teachers in the UK, this is due to a possibility of bias within the sample, in which only those who are comfortable and confident, or have an interest with the menstrual cycle were willing to participate in the survey. Thus, our results could be influenced somewhat by this when asked how comfortable and confident they are teaching menstrual education, and possibly skew the prevalence of menstrual cycle education as we have captured those that have an interest in this area, are aware or provide lessons. Despite this being a limitation, the subsequent effect on the results could mean the prevalence of education is lower than reported in the present study and that teachers are less comfortable and confident delivering menstrual education than what has been discovered. Additional limitations are associated with data collected from surveys as opposed to information rich data that can be achieved through interviews. A balance is required between sample size, which is increased and from a representative population when using surveys. Based on a confidence level of 95 and 5% margin error, it was determined that a minimum of 300 responses was required from the teaching population in the UK. This was exceeded to reduce the margin error to 3% which was deemed acceptable given the novelty of the data collection and view to inform later follow up interviews and further research. The data collected is also from a large proportion of counties/authorities in each country, however without purposeful sampling it cannot be guaranteed that all economic and cultural factors are captured, further work should ensure that equal responses are acquired from differing economic and cultural areas. Such limitations detailed here are common amongst self-reported survey responses, but it should not take away the outcomes we have established, given this is one of the few studies globally to detail such information on menstrual cycle education in schools.

In summary, the findings of this study highlight the urgent need to address menstrual cycle education in schools across the UK, for both boys and girls. Fundamentally, support is required for teachers to increase confidence and knowledge to improve delivery of menstrual education. This can be achieved through provision of teaching resources, training and timetabling; consequently, delivering lessons to pupils at a younger age and on more than one occasion. Training should be provided to all teachers, male and female, to ensure they have an awareness of the menstrual cycle and the potential impact on attendance, attitude and confidence and participation in physical activity to prevent the menstrual cycle having a negative impact on achievement and wellbeing. Teachers should address the importance of menstrual cycle regularity, abnormal uterine bleeding and other menstrual-related disorders within lessons and help girls manage their menstrual symptoms. This type of information is vital for girls to manage their menstrual cycle and seek appropriate help where needed and reduce the potential negative impact of the menstrual cycle.

This paper is a call to action to improve menstrual education for girls and boys across the UK. It is advocated this should be achieved through:

– Time made available for delivery, particularly to increase the number of lessons young people receive, the regularity of teaching and ensuring age-appropriate lessons throughout all stages of the curriculum.
– Resources provided for teachers to deliver information relating to emotion, social and physical aspects of the menstrual cycle in addition to biology of the reproductive system.
– Support provided to teachers in the form of training, receiving online professional development through e-learning and/or webinar(s).
– Development of peer support groups for young people within schools.
– Reframing of the narrative, addressing historical societal constructs.

## Data Availability Statement

The raw data supporting the conclusions of this article will be made available by the authors, without undue reservation.

## Ethics Statement

The studies involving human participants were reviewed and approved by Swansea University. The patients/participants provided their written informed consent to participate in this study.

## Author Contributions

NB, RW, GB, JP, and LF have contributed to all aspects of the reported article. All authors contributed to the article and approved the submitted version.

## Conflict of Interest

Author GB was employed by Orreco Ltd. The remaining authors declare that the research was conducted in the absence of any commercial or financial relationships that could be construed as a potential conflict of interest.

## Publisher's Note

All claims expressed in this article are solely those of the authors and do not necessarily represent those of their affiliated organizations, or those of the publisher, the editors and the reviewers. Any product that may be evaluated in this article, or claim that may be made by its manufacturer, is not guaranteed or endorsed by the publisher.
