# Supplementary material for: Teachers' Perceptions and Experiences of Menstrual Cycle Education and Support in UK Schools
Source: Front Glob Womens Health. 2022 Feb 14;3:827365. doi: 10.3389/fgwh.2022.827365 (PMC8882726; doi:10.3389/fgwh.2022.827365)
Supplement: Supplementary file 1 [file Table_1.DOCX]

Supplementary material:

Comparison of school year groups across England, Wales, Scotland and Northern Ireland

|  | England | Wales | Scotland | Northern Ireland |
| --- | --- | --- | --- | --- |
| School years | Reception  Year 1  Year 2  Year 3  Year 4  Year 5  Year 6  Year 7  Year 8  Year 9  Year 10  Year 11 | Reception  Year 1  Year 2  Year 3  Year 4  Year 5  Year 5  Year 6  Year 7  Year 8  Year 9  Year 10 | Nursery/Early level  P1  P2  P3  P4  P5  P6  P7  S1  S2  S3  S4 | P1  P2  P3  P4  P5  P6  P7  P8  P9  P10  P11  P12 |

Adapted from UK Comparison table of school year groups across the UK (April 2020)

<https://www.raf-ff.org.uk/wp-content/uploads/2020/06/UK-school-year-comparison-table-2020-plus-devolved-state-edu-comparison-table.pdf> [accessed 12.11.21]

Copy of survey tool, without logic which was applied for completion to avoid any irrelevant questions being answered by participants

1 I confirm that I have read and understood the information sheet dated 01/11/2020, version number 1.1 and that I have the opportunity to contact members of the research team to ask questions. I understand that my participation is voluntary, and the questionnaire responses are completely anonymous, even to the research team. I understand that my responses for each question are automatically saved when I answer them, and no identifiable information is recorded in this study. I understand that I am unable to withdraw my data once I have provided a response. I understand that data obtained may be looked at by responsible individuals from Swansea University or from regulatory authorities where it is relevant to my taking part in research. I give permission for these individuals to have access to these records. I understand that data I provide may be used in reports and academic publications in anonymous fashion. Top of Form

- I agree to take part in the above study

p. 2 Menstrual cycle education in schools

Top of Form

2 Please select which country your school is in:

Top of Form

- England
- Wales
- Scotland

Northern Ireland

a Please select which county/local authority your school is in (please select multiple if working at different schools):

Top of Form

- Anglesey
- Brecknockshire
- Caernarfonshire
- Cardiganshire
- Carmarthenshire
- Clwyd
- Denbigshire
- Dyfed
- Flintshire
- Glamorgan
- Gwent
- Gwynedd
- Merionethshire
- Mid Glamorgan
- Monmouthshire
- Montgomeryshire
- Pembrokeshire
- Powys
- Radnorshire
- South Glamorgan
- West Glamorgan
- Prefer not to say

b Please select which county/local authority your school is in (please select multiple if working at different schools):

- Bedfordshire
- Berkshire
- Bristol
- Buckinghamshire
- Cambridgeshire
- Cheshire
- Cornwall
- County Durham
- Cumberland
- Derbyshire
- Devon
- Dorset
- Essex
- Gloucestershire
- Hampshire
- Herefordshire
- Hertfordshire
- Huntingdonshire
- Kent
- Lancashire
- Leicestershire
- Lincolnshire
- Middlesex
- Norfolk
- Northamptonshire
- Northumberland
- Nottinghamshire
- Oxfordshire
- Shropshire
- Somerset
- Staffordshire
- Suffolk
- Surrey
- Sussex
- Warwickshire
- West Midlands
- Wiltshire
- Worcestershire
- Yorkshire
- Other
- Prefer not to say

Top of Form

c Please select which county/local authority your school is in (please select multiple if working at different schools):

- Derry/Londonderry
- Tyrone
- Fermanagh
- Armagh
- Antrim
- Down
- Prefer not to say

Top of Form

d Please select which county/local authority your school is in (please select multiple if working at different schools):

- Aberdeen City Council
- Aberdeenshire Council
- Angus Council
- Argyll and Bute Council
- City of Edinburgh Council
- City of Glasgow Council
- Clackmannanshire Council
- Comhairle nan Eilean Siar (Western Isles Council)
- Dumfries and Galloway Council
- Dundee City Council
- East Ayrshire Council
- East Dunbartonshire Council
- East Lothian Council
- East Renfrewshire Council
- Falkirk Council
- Fife Council
- Highland Council
- Inverclyde Council
- Midlothian Council
- Moray Council
- North Ayrshire Council
- North Lanarkshire Council
- Orkney Islands Council
- Perth and Kinross Council
- Renfrewshire Council
- Scottish Borders Council
- Shetland Islands Council
- South Ayrshire Council
- South Lanarkshire Council
- Stirling Council
- West Dunbartonshire Council
- West Lothian Council
- Prefer not to say

Top of Form

e What year/level(s) do you teach, please select from below:

- Reception
- Year 1
- Year 2
- Year 3
- Year 4
- Year 5
- Year 6
- Year 7
- Year 8
- Year 9
- Year 10
- Year 11

Top of Form

f What year/level(s) do you teach, please select from below:

- P1 (early level)
- P2 (first level)
- P3 (first level)
- P4 (first level)
- P5 (second level)
- P6 (second level)
- P7 (second level)
- S1 (third/fourth level)
- S2 (third/fourth level)
- S3 (third/fourth level)
- S5 (senior phase)
- S6 (senior phase)

Top of Form

g What year/level(s) do you teach, please select from below:

- Year 1
- Year 2
- Year 3
- Year 4
- Year 5
- Year 6
- Year 7
- Year 8
- Year 9
- Year 10
- Year 11
- Year 12

Top of Form

h Following the introduction of the PSHE curriculum in September 2020, is education relating to the menstrual cycle provided for you to teach?

Top of Form

- Yes
- No
- Don't know

i Will information and delivery of education about the menstrual cycle be different from any education previously delivered in your school?

Top of Form

- Yes
- No

ii Has any information/guidance been provided to teachers to start delivering this as part of the new curriculum?

Top of Form

- Yes
- No

iii Do you feel confident and comfortable delivering this as part of the new curriculum?

- Not at all confident and comfortable
- Somewhat confident and comfortable
- Confident and comfortable
- Extremely confident and comfortable

Top of Form

a Please describe the reasoning for your previous response

Top of Form

3 Please select which applies to the school you teach at:

Top of Form

- State (LA)
- State (academy)
- Independent

4 What is your gender?

Top of Form

- Male
- Female
- Prefer not to disclose

Other

a If you selected Other, please specify:

Top of Form

5 Which category includes your age?

Top of Form

- <21
- 21-29
- 30-39
- 40-49
- 50-59
- 60 or older

6 How many years have you been teaching?

Top of Form

- <1yr
- 1-3yrs
- 4-9yrs
- 10+yrs

7 What subject do you teach?

Top of Form

- Mixed (primary/early/first level)
- Maths
- Science
- English
- Geography
- History
- PE
- Design & Technology
- IT
- Art
- Music
- Modern foreign languages
- Multiple subjects
- Class contact cover (CCC)
- Senior leader
- PSHE
- Other

a If you selected Other, please specify:

Top of Form

8 Do you think the menstrual cycle can have an impact on any areas of girls’ performance in school? Please tick the areas you think it affects:

Top of Form

- Exam results
- Learning
- Attendance
- Physical activity/PE
- Attitude/behaviour
- Confidence
- Other

a If you selected Other, please specify:

Top of Form

9 Are menstrual products provided for free within your school?

Top of Form

- Yes
- No
- Don't know

a Please provide any further comments in relation to access to menstrual products within your school

Top of Form

10 Are lessons/education provided on the menstrual cycle at your current school?

Top of Form

- Yes
- No
- Don't know

a What are the reasons/barriers which are preventing education of the menstrual cycle being provided at your school?

Top of Form

b What is taught as part of the education on the menstrual cycle at your school? (please select multiple options if applicable)

Top of Form

- Biology of the menstrual cycle
- What is a regular menstrual cycle and symptoms
- What is considered irregular cycles and symptoms
- What to do if you think your cycle is irregular
- How to manage symptoms
- Scenarios and examples of managing your menstrual cycle e.g. lived experiences
- Menstrual products
- Benefits of exercise for the menstrual cycle & symptoms
- None
- Other

i If you selected Other, please specify:

Top of Form

c Are the lessons/education within a specific subject? Please specify

Top of Form

d Are the lessons/education part of the curriculum?

Top of Form

- Yes
- No
- Don't know

e Within one academic year, how many lessons cover education of the menstrual cycle?

Top of Form

- 1
- 2
- 3
- 4
- 5+
- Don't know

f In what year/level is education on the menstrual cycle provided at your school? Please select as appropriate (select more than 1 option where applicable)

Top of Form

- <year 3
- year 4
- year 5
- year 6
- year 7
- year 8
- year 9
- year 10
- year 11
- <P4
- P5
- P6
- P7
- S1
- S2
- S3
- S4
- S5

g Who are the lessons delivered to?

Top of Form

Edit question

Bottom of Form

Question actions

- Girls and boys together
- Girls alone
- Boys alone
- Other

i If you selected Other, please specify:

Top of Form

h Are parents involved in any of the education, such as through the sharing of at-home resources/parents information events?

Top of Form

Edit question

Bottom of Form

Question actions

- Yes
- No
- Don't know
- Dependent on level/year group

i Are there any reasons menstrual cycle education is not provided? Multiple options are available to select.

- Don't know
- Not enough time
- Not enough support/resources
- Not relevant
- Other

Top of Form

i If you selected Other, please specify:

Top of Form

j Are there any barriers preventing increased or a different approach to menstrual cycle education being provided? Please specify

Top of Form

k Have you delivered any lessons on the menstrual cycle at your current school?

Top of Form

- Yes
- No

i Were you provided with content to deliver on the menstrual cycle?

Top of Form

- Yes
- No

ii How comfortable do you feel teaching information about the menstrual cycle?

- Very uncomfortable
- Uncomfortable
- Neutral
- Comfortable
- Very comfortable

Top of Form

iii Do you feel supported when teaching the menstrual cycle by other colleagues?

Top of Form

- Yes
- No
- Sometimes

iv Do you teach the science (e.g. biology of the reproductive system and hormones) of the menstrual cycle?

Top of Form

- Yes
- No

11 How confident do you feel in your knowledge about the menstrual cycle and the effect on health (e.g. bone health, mental health)

- Not confident at all
- Some what confident
- Confident
- Extremely confident

Top of Form

12 How confident do you feel talking about the menstrual cycle?

- Not confident at all
- Some what confident
- Confident
- Extremely confident

Top of Form

13 How confident do you feel providing advice about the menstrual cycle

- Not confident at all
- Some what confident
- Confident
- Extremely confident

Top of Form

14 Is there anything that would help improve menstrual cycle education being provided? Including any tasks and resources that would help to embed menstrual cycle education within teaching and the school environment?

Top of Form

15 Do you feel it would be beneficial to receive training to help teach about the menstrual cycle?

Top of Form

- Yes
- No

a What format would you like to receive the training in, multiple options are available to select:

- e-learning/webinar
- INSET training from an external professional
- INSET training delivered internally
- Resources to read
- External course
- Other

Top of Form

i If you selected Other, please specify:

Top of Form

16 What is the policy on girls and sport whilst on their periods at your current school?

Top of Form

- We don’t have a policy
- We have a policy
- Don’t know

a Please provide details of the policy you have

Top of Form

17 Are girls encouraged to do sport or is participation altered at your current school?

- Encouraged to participate in sport
- Option to not participate
- Alternative option to exercise provided
- Don’t know

Top of Form

18 Does the menstrual cycle have an impact on girls’ participation who are usually engaged with physical activity? (Extreme impact describes most pubescent girls stopping or reducing their physical activity as a result of their menstrual cycle)

- No impact at all
- Moderate impact
- Majority impact
- Extreme impact
- Don't know

Top of Form

19 Does the menstrual cycle have an impact on girls’ participation who are usually disengaged with physical activity? (Extreme impact describes most pubescent girls stopping or reducing their physical activity as a result of their menstrual cycle)

- No impact at all
- Moderate impact
- Majority impact
- Extreme impact
- Don't know

Top of Form

20 Do you provide any information to girls regarding the benefits of exercise for the management of symptoms associated with the menstrual cycle?

Top of Form

- Yes
- No
- Don't know

21 Do you provide any guidance on menstrual products to help continue participating in sport/physical activity?

Top of Form

- Yes
- No
- Don't know

22 Do you see any performance impacts for girls as a result of their menstrual cycle in physical education/activity?

Top of Form

- Yes
- No
- Don't know

a Please provide detail on the performance impact for girls in physical education/activity

Top of Form

23 Do you have any further comments you would like to add about menstrual cycle education in school?

Top of Form

p. 3 Thank you

Top of Form

The research team would like to sincerely thank you for your time and participation in this UK wide survey.

Please share with all the teachers you know, from anywhere in the UK!

Top of Form

Bottom of Form
